# Spectrum of genetic variants associated with maple syrup urine disease in the Middle East, North Africa, and Türkiye (MENAT): a systematic review

**DOI:** 10.1186/s12920-025-02083-x

**Published:** 2025-03-13

**Authors:** Salma Younes, Razan Elkahlout, Houda Kilani, Sarah Okashah, Hussain Al Sharshani, Zoulikha Rezoug, Hatem Zayed, Nader Al-Dewik

**Affiliations:** 1 Department of Biomedical Science, College of Health Sciences, Qatar University, Doha, Qatar; 2 Department of Research, Women Wellness and Research Center (WWRC), Hamad Medical Corporation (HMC), Doha, Qatar

**Keywords:** Maple syrup urine disease (MSUD), Genetic variants, Genotype-phenotype correlations, MENA

## Abstract

**Background:**

Maple syrup urine disease (MSUD) is a hereditary metabolic disorder caused by a deficiency in the branched-chain α-keto acid dehydrogenase (BCKD) enzymatic complex. The Middle East and North Africa, and Türkiye (MENAT) region has witnessed a significant rise in the prevalence of MSUD due to high rates of consanguinity. Despite numerous genetic association studies, the complex relationships between genotype and phenotype in MSUD remain elusive.

**Aim:**

This study aimed to systematically review the variants significantly associated with MSUD in the MENAT region.

**Methods:**

We systematically searched four literature databases (PubMed, Scopus, Web of Science, and Science Direct) from inception until December 2023 to gather all reported genetic data pertaining to MSUD in the MENAT region. Quality assessment and data extraction were diligently performed by a team of six investigators.

**Results:**

A total of 16 studies, involving patients, were included in this systematic review. Among them, 211 patients presented with 105 variants located within genes known to be associated with MSUD. The majority of the identified MSUD variants were found in *BCKDHA* (38%), followed by *BCKDHB* (38%), *DBT* (23%), and *PPM1K* (1%). Notably, 77% of the captured variants were unique to the MENAT region.

**Conclusion:**

Our systematic review reveals a distinctive genetic and clinical susceptibility profile of MSUD among individuals from the MENAT region. These findings highlight the importance of understanding the specific genetic landscape of MSUD in this population. Further research is warranted to elucidate the complex genotype-phenotype relationships in MSUD in the MENAT region.

**Supplementary Information:**

The online version contains supplementary material available at 10.1186/s12920-025-02083-x.

## Introduction

Maple syrup urine disease (MSUD, MIM #248600) is a recessively inherited inborn error of metabolism characterized by a deficiency of the branched-chain alpha-keto acid dehydrogenase (BCKDH) complex that results in accumulation of branched-chain amino acids (BCAA) including leucine, isoleucine, and valine [1]. This accumulation can lead to severe symptoms and complications, including neurological symptoms, growth failure, and death if left untreated [2]. Patients with MSUD show variable degrees of enzyme deficiency leading to several distinct phenotypes [3]. The types include severe (classic), intermediate, intermittent, thiamine responsive, and E3-deficiency [4], among which classic MSUD is considered the most common and most severe form of the disorder [5, 6].

MSUD is estimated to affect approximately 1 in 185,000 infants worldwide [7]. The incidence varies across populations, with higher rates observed in populations with a high rate of consanguineous marriages [6, 8]. In the Middle East and North Africa, and Türkiye (MENAT) region, the exact incidence rate of MSUD is not well known but is believed to be underdiagnosed due to limited access to diagnostic testing or lack of awareness among healthcare providers. However, according to the few reports in the region, incidence rates were estimated to be 1/21,490 live births in Saudi Arabia [9], 1/13,716 in Tunisia [10], 1/18,180 in Tehran, 1/26,714 in Mazandaran, and 1/21,303 in Fars [11]. MSUD has also been recognized as the most frequently occurring organic Acidemia in Turkey [12].

The MENAT region is characterized by a diverse ethnic landscape with high genetic heterogeneity due to historical and cultural factors, including consanguineous marriages that are common in many Arab countries [13], which makes the genome architecture of these populations unique in terms of susceptibility to various diseases, including both Mendelian and complex diseases [14–25]. Over the past decade, researchers have started to sequence Arab genomes through national projects in hopes of defining disease-associated genetic variants for gene disorders in Arabs and to establish meaningful genotype–phenotype correlations. Starting with Saudi Arabia [26], followed by Qatar [27], and currently the United Arab Emirates [28], these countries are establishing their 1000 Genomes Projects. With the rising incidence rates of MSUD in the MENAT region, there is a growing interest in understanding the genetic architecture that may render populations in this region susceptible to MSUD. Several pathogenic variants causing MSUD have already been described in the *BCKDHA*, *BCKDHB*, and *DBT* genes [3, 7, 29, 30], with over 300 variants identified in the Human Gene Mutation Database (HGMD) and ClinVar. However, genotype-phenotype correlations remain unclear, and there has been relatively little attention devoted to comprehensively investigating genetic variants associated with MSUD in the MENAT region, creating a knowledge gap.

Therefore, the aim of this study is to systematically review the current evidence on genetic variants associated with MSUD in the MENAT region. This research is crucial for identifying potential MSUD-associated genetic variants among MENAT populations, improving premarital genetic counseling, and ultimately enhancing health outcomes and quality of life for patients with MSUD in the region. By filling the knowledge gap and establishing meaningful genotype-phenotype correlations, this study may contribute to better understanding and management of MSUD in the MENAT region.

## Methods

**Fig. 1:**
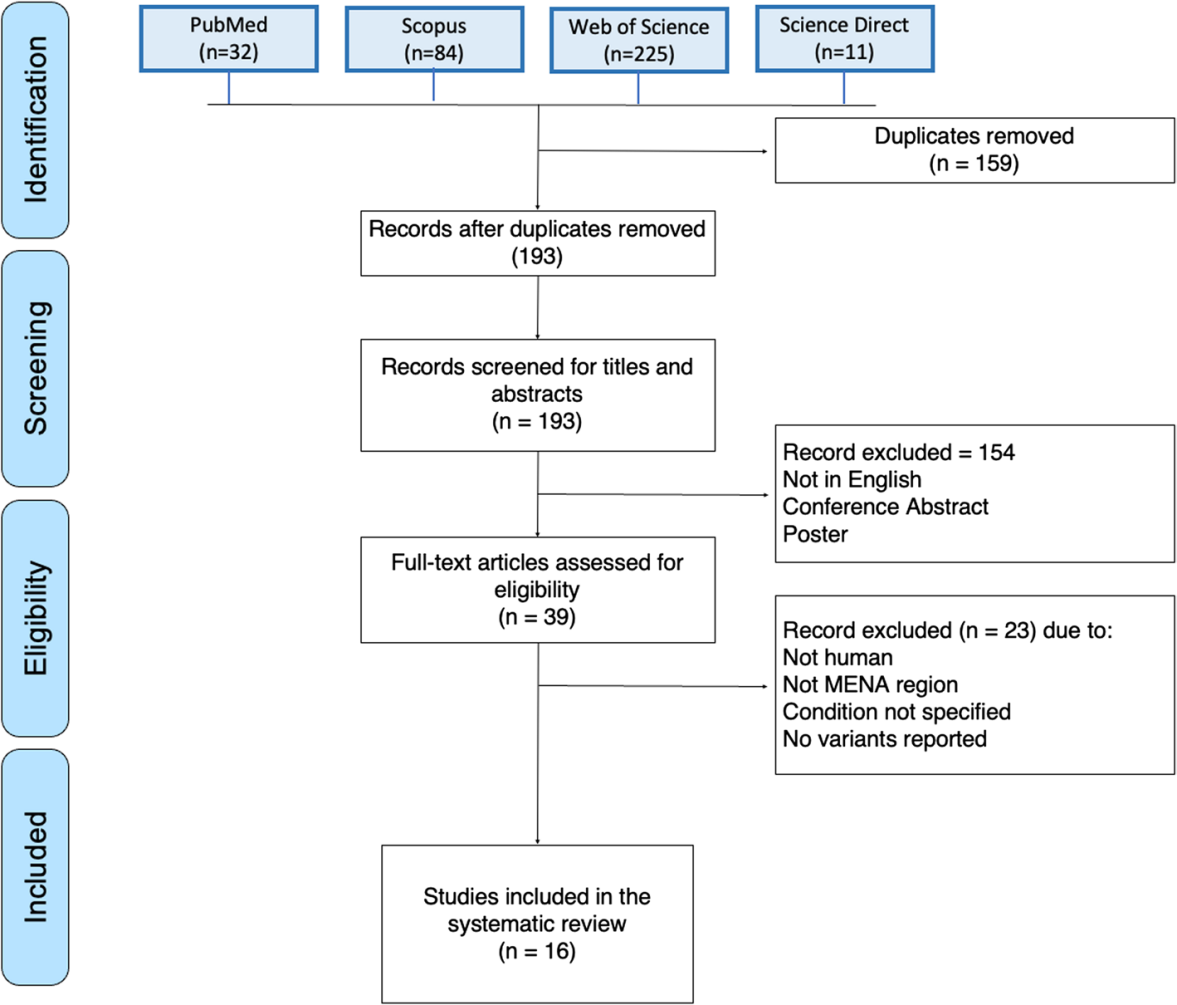
PRISMA flow chart depicting the systematic review's study selection process. The chart provides a visual representation of the systematic review's selection process. A total of 39 full-text articles were evaluated for eligibility. Following rigorous assessment, 16 studies that met the inclusion criteria were included

The present systematic review was developed and executed in accordance with the Preferred Reporting Items for Systematic Reviews and Meta-analyses (PRISMA) guidelines [31] (Fig. 1, Table S1), to ensure that it was as rigorous as possible.

The described search method and selection strategy was used to identify studies investigating the effect of MSUD-associated genetic variants on MSUD risk among patients residing in the MENAT region.

### Search strategy

We systematically searched four literature databases (PubMed, Science Direct, Web of Science, Scopus) to collect information on MSUD-associated pathogenic variants in the MENAT region from inception until December 2023. In addition, cross-referencing from the bibliographies of all retrieved articles (citation tracking) was performed. We utilized several Boolean operators and search strings for the different electronic database searches (Table S2).

To gain a better understanding of the ethnic distribution of the captured variants and whether they are distinctive to populations in the MENAT region, we individually searched the captured variants in the following databases: ClinVar (https://www.ncbi.nlm.nih.gov/clinvar/), dbSNP (https://www.ncbi.nlm.nih.gov/snp/), Human Genome Mutation Database (HGMD) (http://www.hgmd.cf.ac.uk/ac/index.php), PubMed, and Google Scholar.

### Study selection

The inclusion and exclusion criteria were developed using a PECO(T) [participants, exposure, comparator, outcome(s), and type of study] structure. However, as we are looking for observational studies, we only considered participants, exposure, and the outcome of interest. For inclusion, studies had to meet all the following criteria: 1) Participants: patients residing in the MENAT region clinically diagnosed with MSUD. 2) Studies reporting pathogenic variants significantly associated with MSUD (*P* < 0.05). 3) Outcome: MSUD, with diagnosis confirmed by serum BCAAs or genetic analyses. 4) Type of study: no study type limits were applied. Articles published in peer-reviewed journals were included. We excluded duplicate publications, studies on animal subjects, studies conducted on non-Arabs, case series, review articles, and articles not in the English language. The PRISMA flow chart for study selection is shown in Fig. 1.

Six scientists (HK, HS, SY, RK, HZ and NA) worked independently in identifying, screening, and performing the quality control of the extracted data from the four literature databases mentioned above. All citations were exported to Endnote X9, and duplicate citations were removed. Articles were screened in two stages: (1) the first stage involved performing the initial screening of the titles and abstracts and assessing relevance for the scope of the systematic review according to our inclusion/exclusion criteria, (2) the second stage involved retrieval of the full text of each potentially relevant study and screening for content to decide on its inclusion. Articles for which full-text articles could not be retrieved, or only abstracts were available, were reviewed for content and were included if they met the inclusion criteria. Points of discrepancies were resolved through discussion between HK, HS, SY, and RK further points of debate were further resolved with the HZ and NA until a consensus was reached.

### Data extraction and analysis

The following information was extracted and recorded: genes associated with MSUD, variants including their nucleotide change and their protein change, country or origin, number of screened patients in each study, number of MSUD patients with reported variants, severity, consanguinity status, and zygosity status. A thorough search was conducted in public databases (ClinVar and dbSNP) for any missing information.

Furthermore, in order to assess if the variants are unique or shared between the MENAT population and other ethnicities, we have searched each variant individually through different databases including ClinVar (https://www.ncbi.nlm.nih.gov/clinvar/), InterVar (https://wintervar.wglab.org/), Google (https://www.ncbi.nlm.nih.gov/clinvar/), Google scholar (https://www.ncbi.nlm.nih.gov/clinvar/), and gnomAD (https://gnomad.broadinstitute.org/) Human Genome Mutation Database (HGMD) (http://www.hgmd.cf.ac.uk/ac/index.php), Exome Variant Server (EVS) (http://evs.gs.washington.edu/EVS/). If any variant is reported in either real patients in a published article or has been submitted to ClinVar by the clinical laboratory, or reported in the population database (gnomAD), it will be considered as shared variant. Data about shared variants such as the other population reported they are reported in, and their allele frequency and zygosity in are collected. If the variant is totally absent from the mentioned databases, it will be considered as a unique variant to the MENAT region.

To further understand the pathogenic role of the unique variants, we have searched them into using the free online tools PolyPhen and InterVar, which is an automated database that uses AMP-ACMG guidelines 2015 to provide a pathogenic score for the enquired variants.

### Quality assessment

Quality assessment was performed using the assessment tools of the National Heart, Lung, and Blood Institute of the National Institutes of Health (NIH): (1) the NIH Quality Assessment tool for Observational Cohort and Cross-Sectional Studies; and (2) the NIH Quality Assessment tool for Case Series Studies [32]. The NIH tools categorize studies as either good, fair, or poor. Table S4 summarizes the quality assessments of the 16 studies included in this systematic review.

## Results

In this study, we retrieved a total of 351 studies, of which 193 remained after removing duplicates (Fig. 1). After excluding 180 citations due to irrelevant information and/or inability to capture all necessary information, 16 articles were selected for the systematic review (Table 1, Table S3, and Table S4) [3, 9, 12, 33–45]. 211 MSUD patients were confirmed by molecular diagnosis to present at least one variant associated with MSUD (Table 1).

**Table 1:** Summary of patients’ demographics and findings captured from 16 studies

Demographics	*N*	%
*Variants collected *	105	
Unique variants	81	77%
Shared variant	24	23%
Patients screened	4142	
Patients with variants	211	5%
*Number of patients with variants by country*		
Iran	57	27.0%
Turkey	50	23.7%
Kingdom Saudi Arabia (KSA)	50	23.7%
Egypt	36	17.1%
Lebanon	4	1.9%
Jordan	10	4.7%
Tunisia	3	1.4%
Iraq	1	0.5%
*Consanguinity in patients with variants *		
Yes	153	72.5%
Not specified	51	24.2%
No	7	3.3%
*Patients' zygosity status*		
Homozygous	190	90.0%
Compound heterozygous	21	10.0%
*Phenotype severity*		
Severe (classic)	142	67.3%
Moderate/Mild/Intermediate	23	10.9%
Not reported/Unknown	46	21.8%

We identified 105 variants in 211 MSUD patients from eight countries in the MENAT region, of which 77% were unique to the MENAT region (Table 2). The majority of patients with MSUD variants were found to have consanguineous parents (72.5%), and most of them had a homozygous genotype (90%). The severe phenotype was present in 67.3% of all patients, with moderate, mild, and intermediate phenotypes being less common, at approximately 10.9%. The cases where disease severity was not reported made up 21.8%.

**Table 2:** Variants captured among MSUD patients in the MENAT region

Gene	Nucleotide change	Protein change	Zygosity	Variant type	Phenotype	Total number of patients screened	Number of patients reported with the variant	Country of origin	Consanguinity	Ref.
*BCKDHA*	c.488_1167+3del	p.Val163Glyfs*6	HO	Frameshift	Intermediate	5	1	Lebanon	Yes	[33]
*BCKDHA*	c.409G>A†	p.Glu137Lys	HO	Missense	NR	52	1	KSA	Yes	[9]
*BCKDHA*	c.660_663delGTAC	p.Tyr221Glnfs*108*	HO	Frameshift	Severe	52	1	KSA	Yes	[9]
*BCKDHA*	c.809G>A†	p.Ala270Thr	HO	Missense	Intermediate	52	2	KSA	Yes	[9]
*BCKDHA*	c.896A>C†	p.Asp299Ala	HO	Missense	Severe	52	1	KSA	Yes	[9]
*BCKDHA*	c.1270C>T	p.Gln424Ter	HO	Nonsense	Severe	52	1	KSA	Yes	[9]
*BCKDHA*	c.143delT	p.Leu48ArgfsX14	HO	Frameshift	Severe	20, 40	1, 1	Iran, Iran	Yes, Yes	[34, 35]
*BCKDHA*	c.(375+1376-1)(884+1885-1)	Splice	HO	Deletion	Severe	20	1	Iran	Yes	[34]
*BCKDHA*	c.375+648_484+520del	p.Gly126ValfsTer3	HO	Frameshift	Severe	1	1	Kurdish Iraq	Yes	[3]
*BCKDHA*	c.702delT	p.Tyr235ThrfsX94	HO	Frameshift	Severe	40, 20	1, 1	Iran, Iran	Yes, Yes	[34, 35]
*BCKDHA*	c.731G>A†	p.Gly244Glu	HO	Missense	Severe	40, 20	1, 1	Iran, Iran	Yes, Yes	[34, 35]
*BCKDHA*	c.1167+1G>T	Splice	HO	Splice site	Severe	40, 20	1, 1	Iran, Iran	Yes, Yes	[34, 35]
*BCKDHA*	c.(375+1_376–1)_(884+1_885–1)del	Splice	CH	Deletion	Severe	40	1	Iran	Yes	[35]
*BCKDHA*	c.355–356Ins7nt	p.D355Dfs	HO	Frameshift	Severe	40	1	Iran	Yes	[35]
*BCKDHA*	c.703delT	Splice	HO	Deletion	Severe	40	1	Iran	Yes	[35]
*BCKDHA*	c.773G>A†	p.Cys258Tyr	HO	Missense	Severe	12, 19	3, 1	Turkey, Turkey	-, Yes	[12, 43]
*BCKDHA*	c.373C>G‡	p.Gln125Glu	HO	Missense	Severe	12, 19	1, 1	Turkey, Turkey	-, Yes	[12, 43]
*BCKDHA*	c.783G>A+784C>A	p.Cys258Ter	CH	Nonsense	Severe	19	1	Turkey	No	[43]
*BCKDHA*	c.919G>A†	p.Arg297His	CH	Missense	Mild	19, 15	1, 1	Turkey, Turkey	No, Yes	[36, 43]
*BCKDHA*	c.982G>A†	p.Ala328Thr	HO	Missense	Mild	19, 15	1, 1	Turkey, Turkey	Yes, Yes	[36, 43]
*BCKDHB*	c.1006G>A*	p.Gly336Ser	HO	Missense	Severe	52	3	KSA	Yes	[9]
*BCKDHA*	c.908_909delTG	p.Phe304Cysfs*36	HO	Frameshift	Severe	9	1	Jordan	Yes	[37]
*BCKDHA*	c.512_512delT	p.Leu171Argfs*159	CH	Frameshift	Severe	33	1	Egypt	-	[38]
*BCKDHA*	c.947_956delGGGCTGTGGC	p.Arg316Glnfs*11	CH	Frameshift	Severe	33	1	Egypt	-	[38]
*BCKDHA*	c.859_866delCGAGGCCC	p.Gly288Valfs*11	CH	Frameshift	Severe	33	1	Egypt	-	[38]
*BCKDHA*	c.205C>T	p.Gln69Ter	HO	Nonsense	Severe	19	1	Turkey	No	[43]
*BCKDHB*	c.716A>G†	p.Glu239Gly	HO	Missense	Severe	3	2	Tunisia	Yes	[39]
*BCKDHB*	c.(343+1_344–1)_(742+1_743–1)del	Splice	CH	Deletion	Severe	40	1	Iran	Yes	[35]
*BCKDHB*	c.92_102del	p.Arg31Glnfs*16	HO	Frameshift	Intermediate	5	1	Lebanon	Yes	[33]
*BCKDHB*	c.197G>C‡	p.Gly66Arg	HO	Missense	Severe	52	3	KSA	Yes	[9]
*BCKDHB*	c.286delGAA	p.Glu96del	HO	Deletion	Severe	52	1	KSA	Yes	[9]
*BCKDHB*	c.1004G>A‡	p.Gly335Asp	HO	Missense	Severe	52	3	KSA	Yes	[9]
*BCKDHB*	c.1145T>C†	p.Cys382Ser	HO	Missense	Severe	52	1	KSA	Yes	[9]
*BCKDHB*	c.833_834insCAC	p. Gly278_Thr279insThr	HO	Insertion	Severe	40, 21	1, 1	Iran, Iran	Yes, Yes	[35] [41]
*BCKDHB*	c.834_836dupCAC	Splice	HO	Duplication	Severe	40	1	Iran	Yes	[35]
*BCKDHB*	c.484A>G†	p.Asn162Asp	HO	Missense	Severe	40	1	Iran	Yes	[35]
*BCKDHB*	c.357delT	p.Leu119>Leufs	HO	Frameshift	Severe	40	1	Iran	Yes	[35]
*BCKDHB*	c.272C>T†	p.Ala91Val	HO	Missense	Severe	19	2	Turkey	Yes	[43]
*BCKDHB*	c.1149T>A	p.Tyr383Stop	HO	Nonsense	Severe	19, 19	3, 2	Turkey, Turkey	Yes, -	[40, 43]
*BCKDHA*	c.116C>A*	p.Pro39His	HO	Missense	Severe	19	1	Turkey	-	[40]
*BCKDHB*	c.688G>T	p.Glu230Ter	HO	Nonsense	Severe	19	1	Turkey	Yes	[43]
*BCKDHB*	c.1015T>C†	p.Ser339Leu	HO	Missense	Severe	19	1	Turkey	Yes	[43]
*BCKDHB*	c.(274+-1_275-1)_(343+-1_344-1)del	Splice	CH	Deletion	Severe	21, 40	1, 1	Iran, Iran	Yes, Yes	[35, 41]
*BCKDHB*	c.1091A>G†	p.Asp364Gly	HO	Missense	Moderate	33	4	Egypt	-	[38]
*BCKDHB*	c.806G>A†	p.Gly269Glu	HO	Missense	Moderate	33	1	Egypt	-	[38]
*BCKDHB*	c.287_287delA	p.Asp97Metfas*133	HO	Frameshift	Moderate	33	2	Egypt	-	[38]
*BCKDHB*	c.908_909insA	p.Asp303Glufs*15	HO	Frameshift	Severe	33	2	Egypt	-	[38]
*DBT*	c.224G>A†	p.Gly75Glu	HO	Missense	Severe	5	2	Lebanon	Yes	[33]
*DBT*	c.1195T>C†	p.Ser399Pro	HO	Missense	Severe	52	1	KSA	Yes	[9]
*DBT*	c.1281+3A>G	Splice	CH	Splice site	NR	52	2	KSA	Yes	[9]
*DBT*	c.562G>T	p.Gly188Trp	HO	Missense	Severe	40	1	Iran	Yes	[35]
*DBT*	c.(363delCT)+(1238T>C)†	p.(Leu121Leufs) + (Ile413Thr)	CH	Frameshift	Severe	40	1	Iran	Yes	[35]
*DBT*	c.(433+1_434–1)_(939+1_940–1)del	Splice	CH	Deletion	Severe	40	1	Iran	Yes	[35]
*DBT*	c.1174A>C ‡	p.Thr392Pro	HO	Missense	Severe	10, 40	1, 1	Iran, Iran	Yes, Yes	[35, 45]
*DBT*	c.85_86insAACG	Splice	HO	Insertion	Severe	40	1	Iran	Yes	[35]
*DBT*	IVS3-1G>A	Splice	HO	Splice site	Severe	12, 19	1, 1	Turkey, Turkey	-, Yes	[12, 43]
*DBT*	c.788T>G†	p.Met263Arg	HO	Missense	Severe	19	2	Turkey	Yes	[43]
*DBT*	c.940-1G>A	p.Ala314-Lys339del	HO	Deletion	Severe	32, 19, 19	1, 1, 1	Turkey, Turkey, Turkey	Yes, Yes, -	[42, 43] [40]
*DBT*	c.1333_1336delAATG	p.Asn445Ter	HO	Nonsense	Severe	3	1	Tunisia	Yes	[39]
*DBT*	c.787A>T†	p.Met263Leu	HO	Missense	Severe	9	2	Jordan	Yes	[37]
*DBT*	c.1202T>C	p.Ile401Thr	HO	Missense	Severe	19	1	Turkey	Yes	[43]
*DBT*	c.61delC	p.Arg21Alafs*12*	HO	Frameshift	NR	52	2	KSA	Yes	[9]
*DBT*	c.939-2A>G	Splice	HO	Splice site	Severe	52	1	KSA	Yes	[9]
*DBT*	IVS8-1G>A+c.1202T>C	Splice	CH	Splice site	Severe	19	1	Turkey	No	[43]
*DBT*	c.1291C>T*	p.Arg431Stop	HO	Nonsense	NR	33, 40	5, 1	Egypt, Iran	- , Yes	[38] [35]
*BCKDHA*	c.1312T>A*	p.Tyr438Asn	HO	Missense	NR	33	4	Egypt	-	[38]
*PPM1K*	c.1A>G	N/A	HO	Start-loss	NR	1	1	Turkey	Yes	[44]
*DBT*	c.241_242delGT	p.Val81Ter	CH	Nonsense	Severe	33	1	Egypt	-	[38]
*BCKDHB*	c.1A>T	p.Met1?*	HO	Nonsense	Severe	52	3	KSA	Yes	[9]
*BCKDHA*	c.288+1G>A*	Splice	HO	Splice site	Severe	40, 20	2, 1	Iran, Iran	Yes, Yes	[35] [34]
*BCKDHB*	c.331C>T*	p.Arg111Ter	HO	Nonsense	Severe	19	1	Turkey	Yes	[43]
*BCKDHA*	c.347A>G	p.Asp116Gly	HO	Missense	Severe	52	1	KSA	Yes	[9]
*BCKDHB*	c.410C>T*	p.Ala137Val	HO	Missense	NR	34, 40	1, 1	Egypt, Iran	-, Yes	[35, 38]
*BCKDHA*	c.452C>T*	p.Thr151Met	HO	Missense	Severe	40, 19	1, 1	Iran, Turkey	Yes, -	[35] [40]
*DBT*	c.74delAT	p.C26Wfs*12*	HO	Frameshift	NR	52	1	KSA	Yes	[9]
*DBT*	c.137A>G	p.Lys46Arg	HO	Missense	Severe	52	3	KSA	Yes	[9]
*BCKDHB*	c.477+1G>A*	Splice	HO	Splice site	Severe	40	1	Iran	Yes	[35]
*BCKDHB*	c.502C>T*	p.Arg168Cys	HO	Missense	Severe	52	2	KSA	Yes	[9]
*BCKDHB*	c.547C>T*†	p.Arg183Trp	HO	Missense	Severe	19	1	Turkey	Yes	[43]
*BCKDHB*	c.564T>A	p.Cys188Stop	HO	Nonsense	Severe	19	1	Turkey	Yes	[43]
*DBT*	c.30G>A	p.Try10Stop	HO	Nonsense	NR	34	1	Egypt	-	[38]
*BCKDHB*	c.574G>A	p.Gly192Arg	HO	Missense	Severe	52	1	KSA	Yes	[9]
*BCKDHB*	c.599C>T*	p.Pro200Leu	HO	Missense	Severe	40	1	Iran	Yes	[35]
*BCKDHB*	c.633+1G>A*	Splice	HO	Splice site	Severe	40, 21	2, 1	Iran, Iran	Yes, Yes	[35] [41]
*BCKDHA*	IVS6-1G>C	Splice	HO	Splice site	Severe	19	1	Turkey	Yes	[43]
*BCKDHA*	c.647-1G>C*	Splice	CH	Splice site	NR	52, 9	1, 3	KSA, Jordan	Yes, No	[9] [37]
*DBT*	c.670G>T	p.Glu224Stop	HO	Nonsense	NR	33	4	Egypt	-	[38]
*BCKDHB*	c.752T>C	p.Val251Ala	HO	Missense	Severe	19, 19	1, 1	Turkey, Turkey	Yes, -	[40, 43]
*BCKDHA*	c.757G>A*	p.Ala253Thr	HO	Missense	Severe	19	1	Turkey	Yes	[43]
*BCKDHB*	c.665A>G	p.Lys222Arg	HO	Missense	Severe	19, 19	1, 1	Turkey, Turkey	Yes, -	[40, 43]
*BCKDHB*	c.817A>C	p.Thr273Pro	HO	Missense	Intermediate	52	8	KSA	Yes	[9]
*BCKDHB*	c.853C>T*	p.Arg285Stop	HO	Nonsense	NR	52, 34, 19, 40, 19	2, 1, 2, 1, 1	KSA, Egypt, Turkey, Iran, Turkey	Yes, - , Yes, Yes, -	[9, 38] [35, 40, 43]
*BCKDHA*	c.859C>T*	p.Arg287Stop	HO	Nonsense	NR	34, 19, 19	1, 1, 1	Egypt, Turkey, Turkey	-, -, Yes	[38, 40] [43]
*BCKDHA*	c.868G>A*	p.Gly290Arg	HO	Missense	Severe	19, 19	1, 1	Turkey, Turkey	-, Yes	[40, 43]
*BCKDHB*	c.508G>T*	p.Arg170Cys	HO	Missense	Severe	40, 21	3, 5	Iran, Iran	Yes, Yes	[35, 41]
*BCKDHA*	c.890G>A	p.Arg297His	HO	Missense	Severe	40, 9	1, 2	Iran, Jordan	Yes, Yes	[35, 37]
*BCKDHA*	c.905A>C*	p.Asp302A1a	HO	Missense	Severe	52	4	KSA	Yes	[9]
*BCKDHB*	c.730T>C	p.Tyr244His	HO	Missense	Severe	40	1	Iran	Yes	[35]
*BCKDHA*	c.940C>T*	p.Arg314Ter	HO	Missense	Severe	52	1	KSA	Yes	[9]
*BCKDHB*	c.970C>T*	p.Arg324Stop	HO	Nonsense	NR	34	2	Egypt	-	[38]
*BCKDHB*	c.988G>A*	p.Glu330Lys	HO	Missense	Severe	40, 21	5, 4	Iran, Iran	Yes, Yes	[35, 41]
*BCKDHB*	c.995C>T	p.Pro332Leu	HO	Missense	NR	34	4	Egypt	-	[38]
*BCKDHA*	c.1251delC	p.Aal418Profs*67	HO	Frameshift	NR	9	1	Jordan	Yes	[37]
*DBT*	c.1057A>T,c.1150A>G*	p.Gly353Ser, p.Gly384Ser	CH	Missense	NR	9	1	Jordan	Yes	[37]
*BCKDHA*	IVS8-2A>G	Splice	HO	Splice site	Severe	19	1	Turkey	Yes	[43]

Homozygous mutations (Zygosity HO) denote instances where both alleles are identical, while Compound Heterozygous mutations (Zygosity CH) involve different alleles. The presence (Consanguinity Yes) or absence (Consanguinity No) of consanguineous relationships is indicated. PolyPhen (Probably damaging†): Predicted to be damaging to protein function. PolyPhen (Possibly damaging ‡): Predicted to possibly be damaging to protein function. Variants marked with an asterisk (*) are considered shared if reported in real patients, published articles, submitted to ClinVar, or present in the population database (gnomAD). Data about shared variants, including their reported populations and allele frequencies, are provided in Table S3 and Fig. 3. Variants without an asterisk are deemed unique to the MENAT region if absent from the mentioned databases and are depicted in Fig. 3. The abbreviation NR denotes Not Reported or Not Applicable

**Fig. 2:**
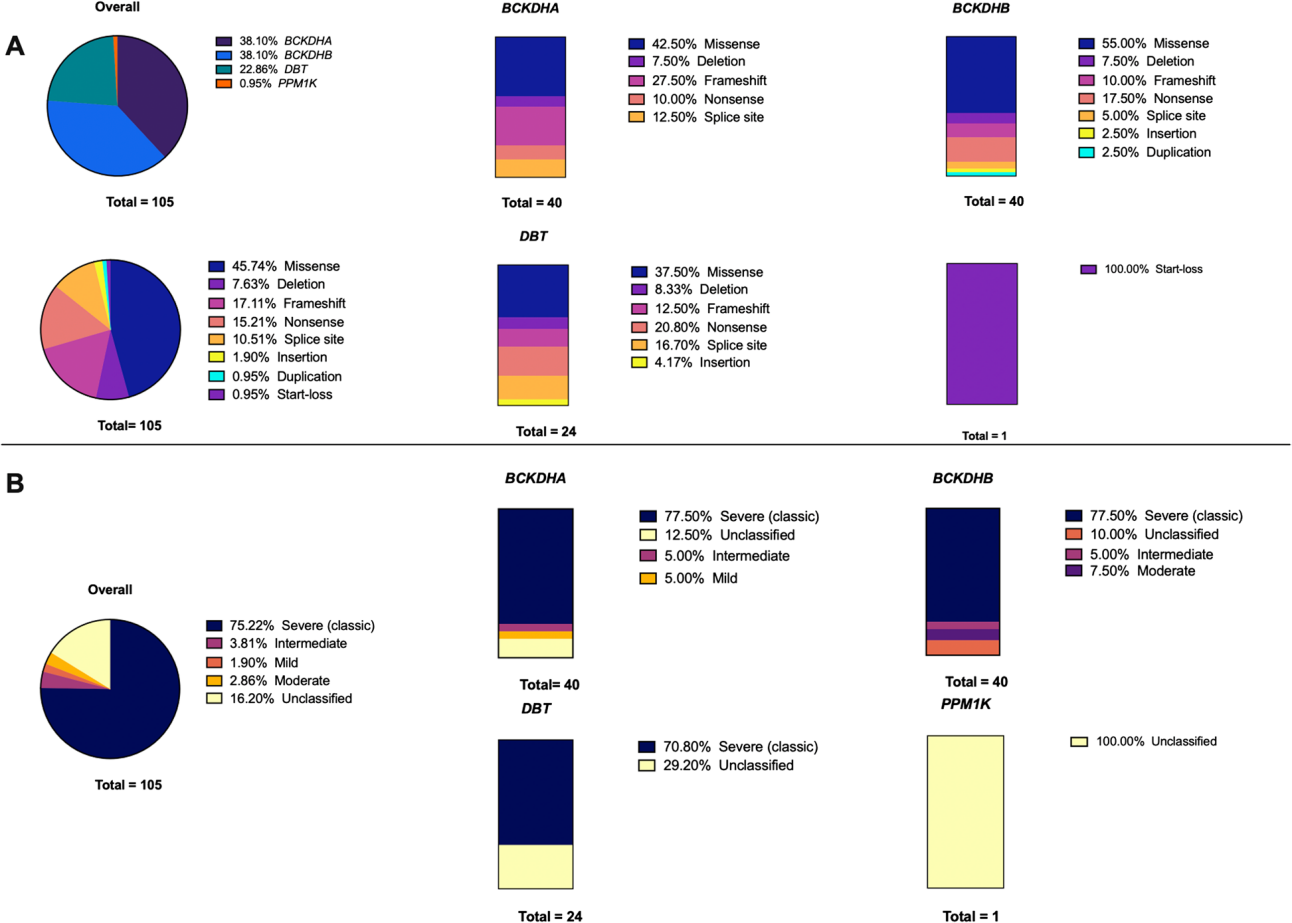
Spectrum of MSUD-associated variants. **A** The implicated genes reported in association with MSUD are classified by the types of variants. **B** Captured variants classified by type of MSUD and the implicated genes

Among the 105 captured variants, (38%) were located in *BCKDHA*, and (38%) were located in *BCKDHB*, these were then followed by variants located in the *DBT* gene (23%), and *PPM1K* (1%) (Fig. 2). Out of the 105 captured variants, 77% of these variants were unique to the MENAT region. Unique variants were identified in patients from Lebanon, Turkey, Jordan, KSA, Iran, Iraq, Egypt, and Tunisia. Some of these unique variants, such as c.919G > A and c.982G > A (*BCKDHA*) in Turkey and c.731G > A (*BCKDHA*) and c.833_834insCAC (*BCKDHB*) in Iran, were reported multiple times in different patients from different studies in the same population.

**Fig. 3:**
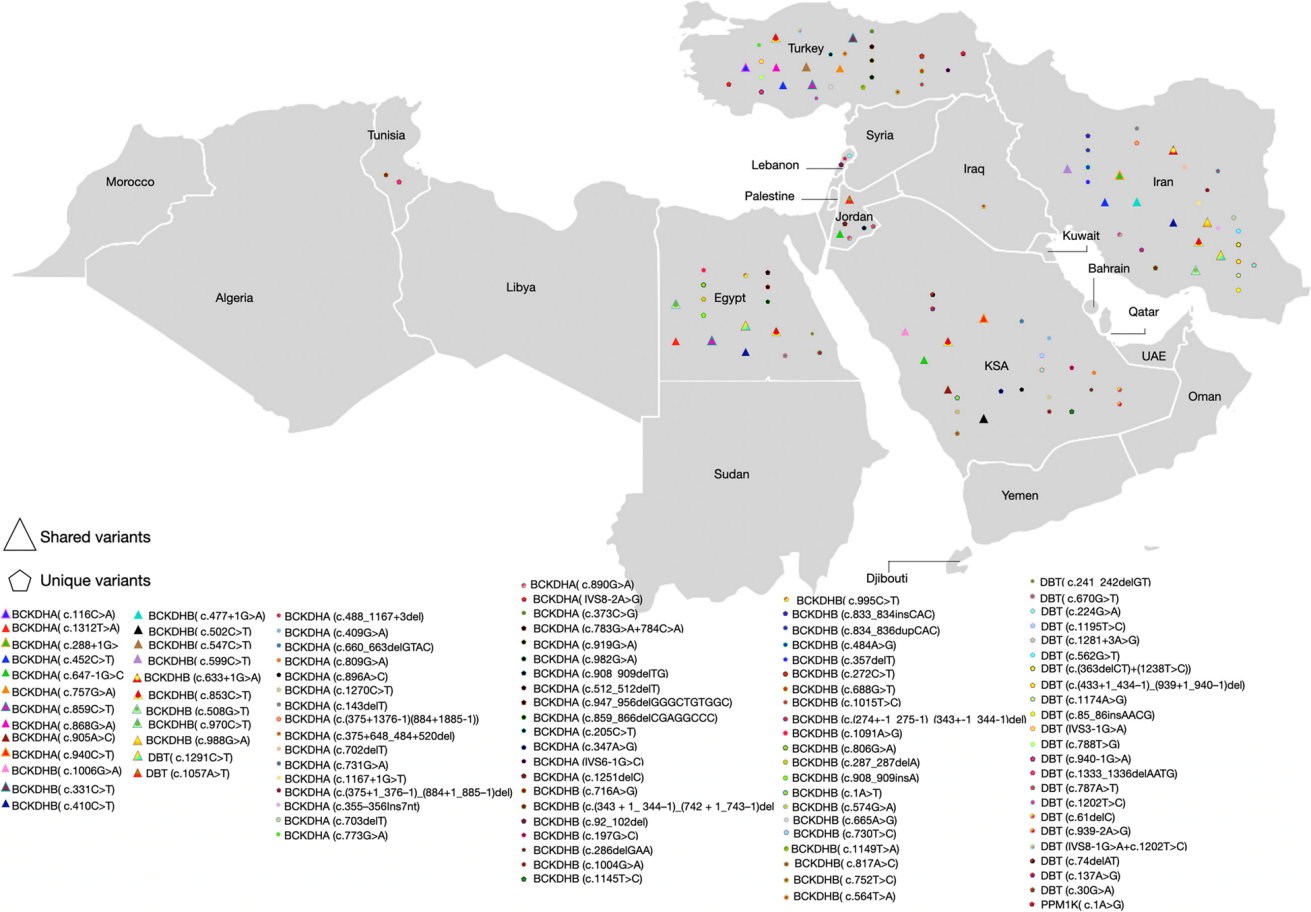
Genetic Landscape of MSUD Variants in the MENAT Population. The map visually presents the distribution of reported MSUD variants within the MENAT population. Triangle-shaped icons denote shared variants, while pentagon-shaped icons represent unique variants. To determine the uniqueness or shared nature of these variants across ethnicities, each variant underwent individual scrutiny through diverse databases including ClinVar, InterVar, Google Scholar, and gnomAD, as well as the Human Genome Mutation Database (HGMD) and Exome Variant Server (EVS). Shared variants, reported in patients or submitted to ClinVar, gnomAD, or published literature, are meticulously recorded with details on other populations reporting them, along with allele frequency and zygosity information. Variants absent from these databases are considered unique to the MENAT region. Abbreviations: KSA - Kingdom Saudi Arabia, HO - Homozygous, CH - Compound Heterozygous, MSUD - Maple Syrup Urine Disease

We also identified 24 variants in *BCKDHA*, *BCKDHB*, *DBT*, and *PPM1K* that were shared with other populations. Some of the shared variants, such as c.1006G > A in *BCKDHB*, c.288 + 1G > A in *BCKDHA*, and c.1291 C > T in *DBT*, were also shared among patients from the MENAT region. The distribution of these variants among the MENAT population is illustrated in Fig. 3 and listed in Table S3.

## Discussion

This is the first systemic review to comprehensively summarize all genetic variants associated with MSUD in the MENAT region. In this systematic review, we captured 105 genetic variants from 16 eligible studies [3, 9, 12, 33–45], comprising 211 patients presented with at least one variant associated with MSUD. We identified 105 variants in four genes (Table 2, Table S3). Among the four genes that have been reported, *BCKDHA (38%)* and *BCKDHB (38%)* were the most frequently affected genes (Fig. 2) among patients with MSUD in the MENAT region, followed by *DBT (23%)*,* and PPM1K (1%). *These findings are in concordance with other studies in Chinese and Indian populations in which the most commonly affected genes were reported to be either *BCKDHA* or *BCKDHB* [46–49], and that the frequency of variants in *BCKDHA* and *BCKDHB* is nearly equal [36, 46, 48]. Nevertheless, other populations, such as Malaysian population, have reported different distribution patterns, in which almost half of the variants were identified in *BCKDHB*, and the other half was distributed closely between *BCKDHA* and *DBT* [50], suggesting that the distribution of variants varies according to ethnicity. In this study, the variant *BCKDHB*,* rs398124599*,* c.853 C > T* was the most frequently reported variant among all variants captured (Table 1), reported five times in four different countries in association with MSUD (Table 2). *BCKDHB*,* rs398124599*,* c.853 C > T* is a nonsense variant that has been reported in other non-Arab ethnicities in association with MSUD and has been reported in ClinVar as Pathogenic/Likely pathogenic/Uncertain significance for MSUD (Table S3).

### Variants unique to the MENAT region

We identified 105 variants associated with MSUD among patients from MENAT countries. Of these variants, 81 (77%) were unique to the MENAT region, with the majority of them being reported in Iran, Turkey, and KSA. Most of the captured unique variants were located in *BCKDHA and BCKDHB* (Table 1).

The high rate of consanguinity in MENAT populations contributes to the increased frequency of homozygous genotypes observed in our study, which in turn leads to a higher prevalence of genetic disorders [51]. Among the homozygous variants, four unique missense variants (c.896 A > C, c.731G > A, c.773G > A, c.373 C > G) were reported in severe/classic MSUD cases. The missense variant c.896 A > C was identified as a novel variant in the original study among the Saudi population and was predicted to be disease-causing and probably damaging. This variant resulted in an amino acid substitution from Aspartic acid to Alanine at position 299 (p.Asp299Ala) [9] (Table 1). In the same Saudi study, nineteen other variants were reported as novel variants associated with MSUD.

The remaining variants that were found to be unique to the MENAT region were located in *BCKDHB* and *DBT* (Table 1). A total of 28 variants located in *BCKDHB* were found to be unique to populations in the MENAT region (Table 1). Among which, one variant (c.688G > T), a homozygous nonsense variant that results in a premature stop codon, was classified as pathogenic in association with MSUD, according to ACMG. A total of 22 variants located in *DBT* were found to be unique to populations in the MENAT region (Table 1). The variant c.85_86ins AACGA is an insertion (c.85_86ins AACG) that leads to a frameshift outcome and premature stop codon (position of a stop codon in WT/Mut CDS 1449/147) and the resulting mRNA will be degraded by nonsense-mediated-decay (NMD). (Table 1). Furthermore, one start-loss mutation was located in the *PPM1K* gene in Turkey. It led to a mild-intermediate MSUD diagnosis [44]. There is only one other reported variant located in *PPM1K* and it was first recorded in Spain in 2013 [52].

The identification of unique variants in MENAT populations highlights the importance of studying genetic diversity across different populations and emphasizes the need for population-specific genetic screening programs to improve diagnosis and management of MSUD. The development of population-specific genetic screening programs could lead to early diagnosis and treatment of MSUD, ultimately reducing the morbidity and mortality associated with this disorder. In addition, the identification of unique variants in MENAT populations could have broader implications for precision medicine, highlighting the importance of considering genetic diversity in the development of targeted therapies for genetic disorders.

### Variants shared with other ethnic groups

In the current systematic review, 24 variants located in *BCKDHA*,* BCKDHB*,* DBT*, and *PPM1K* were found were found to exhibit genotype-phenotype correlations that are shared between populations in the MENAT region and other ethnicities (Table S3).

The majority of variants in the “shared variants” category were found in the *BCKDHB* gene (*n* = 12) (Table S3). Among them is the missense variant rs398124560, which has been identified in MSUD patients from KSA (Table 2). This variant results in the nucleotide change c.1006G > A. It has been linked to severe classic type MSUD in KSA [9] (Table 2) and has also been previously reported in association with MSUD in China [53]. *BCKDHB* variants have been implicated in MSUD and have been extensively studied worldwide, with documented cases in various ethnic groups such as Chinese, Iranian, and Japanese populations (Table S3). *BCKDHA* was the second most prevalent gene in this category, with 10 identified variants linked to MSUD in multiple countries within the MENAT region, as well as in non-Arab countries (Table 2, Table S3). In relation to the *DBT* gene, a total of two variants showed genotype-phenotype correlations that were shared among populations in the MENAT region and other ethnicities (Table S3).

### Distribution of genetic variants in the MENAT region and their respective phenotypes

The identification of MSUD-associated variants in Arab populations play an important role in disease identification, disease management, and genetic counselling. The variant spectrum of MSUD has been assessed in many countries, and the distribution of *BCKDHA*, *BCKDHB*, *DBT*, and *PPM1K* for patients with MSUD was found to vary widely between different populations [50]. Over 500 disease-causing variants have been described in Human Gene Mutation Database (HGMD) (http://www.hgmd.cf.ac.uk/ac/index.php) in *BCKDHA*, *BCKDHB DBT*, *PPM1K* genes. The number of affected people with MSUD within the MENAT region was reported to be higher than the reported prevalence around the world [35]. For example, in Saudi Arabia, the prevalence rate was estimated to be 1 per 21490 birth [9]. This is mainly attributed to consanguinity; the average rates of consanguinity ranges between 40-50% in the Arab world, and these numbers may be up to the level of 60% in some societies, as in the United Arab Emirates and Saudi Arabia [4], hence the rate of autosomal disease is higher in proportion than western countries.

Genotype-phenotype correlations for MSUD severity remain complex and are not fully understood. Although various studies have linked specific gene variants with MSUD severity, consistent associations between variants and phenotypes across populations are lacking. Research on gene variants associated with MSUD has revealed conflicting results, as the same variant often manifests differently across populations. For instance, in an Indian genotype-phenotype study, BCKDHB variants were predominantly associated with classic MSUD phenotypes [49]. However, our findings indicate that BCKDHB variants in the MENAT region present a broader phenotypic spectrum, with patients exhibiting not just classic phenotype, but intermediate and moderate MSUD types (Table S3). For example, the c.92_102del (p.Arg31Glnfs16) variant is linked to an intermediate phenotype in Lebanon, while c.1091 A > G (p.Asp364Gly), c.806G > A (p.Gly269Glu), and c.287_287delA (p.Asp97Metfs*133) in Egypt are associated with moderate MSUD severity. Additionally, the BCKDHB c.817 A > C (p.Thr273Pro) variant in Saudi Arabia is linked to an intermediate phenotype.

Similarly, while *BCKDHA* is commonly associated with classic severe MSUD phenotypes, some studies have observed intermediate MSUD-associated variants in *BCKDHA* as well [54]. Our study identified BCKDHA variants correlating with intermediate and mild phenotypes in Lebanon, Saudi Arabia, and Turkey. Specifically, BCKDHA variants such as c.488_1167 + 3del (p.Val163Glyfs6) in Lebanese patients and c.809G > A (p.Ala270Thr) in Saudi patients are associated with intermediate MSUD, while variants c.919G > A (p.Arg297His) and c.982G > A (p.Ala328Thr) in Turkish patients correlate with mild Type IA MSUD.

Additionally, the *DBT* gene has been linked to classic, intermediate, and thiamine-responsive MSUD phenotypes [55–57]. These findings suggest that even with identical gene variants, phenotypic expression may vary across populations, pointing to the influence of potential secondary factors such as modifier genes or environmental conditions that may interact with primary MSUD mutations to shape clinical outcomes [47]. These modifiers, along with environmental factors, add complexity to genotype-phenotype predictions in MSUD, underscoring the need for further studies focused on these interactions.

This study is the first to systematically summarize all reported MSUD-associated genetic variants in the MENAT region, using stringent criteria and in-depth analysis of each article. Although a meta-analysis was not possible, key findings were presented narratively, which could help identify population-specific founder effects and genotype-phenotype patterns. This research offers critical insights for MSUD diagnosis and prevention, supporting genetic screening methods like carrier testing and prenatal diagnostics in the region. However, limitations include inconsistent MSUD classifications and high variability in study designs, which complicated genotype-phenotype correlation analysis. Few genetic studies focused specifically on MSUD in the MENAT region, leaving knowledge gaps in understanding MSUD genetics within this population. Future research would benefit from standardized MSUD classifications, increased collaboration, and data sharing across studies to improve clarity and support comprehensive analyses of MSUD in diverse populations.

## Conclusions

This systematic review was designed to comprehensively assess all genetic variants significantly associated with MSUD risk in the MENAT region. Our findings indicate that people in the MENAT region have distinct disease susceptibility genotypes that are responsible for MSUD. Although some of the MSUD-associated variants captured in this study have been reported in other ethnic groups, the complex gene-environment interactions allow for enrichment of these genotypes, thus predisposing individuals of these ethnic groups to MSUD. Our study creates a paradigm for future well-controlled epidemiological studies that will allow dissection of the genetic architecture that renders people in the MENAT region susceptible to MSUD and thus may in the future serve as a platform to design a gene panel for early and accurate diagnosis of MSUD. Despite our comprehensive search strategy, the dearth of genetic association studies related to MSUD in the MENAT region suggests a need for additional well-designed genetic association studies to serve as the basis for understanding the genetic architecture that renders Arab populations susceptible to MSUD.

## Supplementary Information

Supplementary Material 1.

Supplementary Material 2.

Supplementary Material 3.

Supplementary Material 4.

## Data Availability

No datasets were generated or analysed during the current study.
