# Supplementary material for: Spectrum of genetic variants associated with maple syrup urine disease in the Middle East, North Africa, and Türkiye (MENAT): a systematic review
Source: BMC Med Genomics. 2025 Mar 13;18:49. doi: 10.1186/s12920-025-02083-x (PMC11905697; doi:10.1186/s12920-025-02083-x)
Supplement: Supplementary file 2 — Supplementary Material 2. [file 12920_2025_2083_MOESM2_ESM.docx]

**Table S2**

| **Database** | **Search Strategy** |
| --- | --- |
| **PubMed** | (MSUD OR Maple syrup urine disease OR Branch Chain alpha keto Acid dehydrogenase deficiency OR BCKDH) AND (Gene OR Genetic OR Mutation OR Variant OR SNP OR polymorphism) AND (MENA OR algeria[mesh] OR egypt[mesh] OR libya[mesh] OR morocco[mesh] OR tunisia[mesh] OR south sudan[mesh] OR sudan[mesh] OR middle east[mesh:noexp] OR bahrain[mesh] OR iraq[mesh] OR jordan[mesh] OR kuwait[mesh] OR lebanon[mesh] OR oman[mesh] OR qatar[mesh] OR saudi arabia[mesh] OR syria[mesh] OR united arab emirates[mesh] OR yemen[mesh] OR algeria*[tw] OR somalia[mesh] OR mauritania[mesh] OR djibouti[mesh] OR arabs[mesh] OR comoros[mesh] OR bahrain*[tw] OR egypt*[tw] OR iraq*[tw] OR jordan*[tw] OR kuwait*[tw] OR lebanon[tw] OR lebanese[tw] OR libanaise[tw] OR yemen*[tw] OR UAE[tw] OR emirat*[tw] OR abu-dhabi[tw] OR dubai[tw] OR libya*[tw] OR morocco[tw] OR moroccan*[tw] OR oman*[tw] OR muscat[tw] OR palestin*[tw] OR gaza[tw] OR west-bank[tw] OR qatar*[tw] OR saudi*[tw] OR KSA[tw] OR syria*[tw] OR tunis*[tw] OR comoros*[tw] OR sudan*[tw] OR arabs[tw] OR arab[tw] OR arabia[tw] OR somali*[tw] OR mauritania*[tw] OR djibouti*[tw] OR levant[tw]) |
| **Scopus** | TITLE-ABS-KEY((MSUD OR Maple syrup urine disease OR Branch Chain alpha keto Acid dehydrogenase deficiency OR BCKDH) AND (Gene OR Genetic OR Mutation OR Variant OR SNP OR polymorphism) AND (lebanon OR lebanese OR algeria* OR bahrain* OR egypt* OR iraq* OR jordan* OR kuwait* OR libanaise OR comoros* OR yemen* OR dubai OR (abu W/2 dhabi) OR UAE OR emirat* OR libya* OR Somalia OR morocco OR moroccan* OR Tunisia OR tunisian OR oman* OR muscat OR palestin* OR gaza OR qatar* OR saudi* OR KSA OR Syria* OR tunis* OR sudan* OR djibouti* OR somali* OR mauritania* OR levant OR arabs OR arab OR arabia)) |
| **Science Direct** | (MSUD OR Maple syrup urine disease OR Branch Chain alpha keto Acid dehydrogenase deficiency OR BCKDH) AND (Gene OR Genetic OR Mutation OR Variant OR SNP OR polymorphism) AND (lebanon OR lebanese OR algeria* OR bahrain* OR egypt* OR iraq* OR jordan* OR kuwait* OR comoros* OR yemen* OR dubai OR (abu W/2 dhabi) OR UAE OR emirat* OR libya* OR Somalia OR morocco OR moroccan* OR Tunisia OR tunisian OR oman* OR muscat OR palestin* OR gaza OR qatar* OR saudi* OR KSA OR Syria* OR tunis* OR sudan* OR djibouti* OR somali* OR mauritania* OR levant OR arabs OR arab OR arabia) |
| **Web of Science** | TS=( MSUD OR Maple syrup urine disease OR Branch Chain alpha keto Acid dehydrogenase deficiency OR BCKDH) AND (Gene OR Genetic OR Mutation OR Variant OR SNP OR polymorphism) AND (algeria* or bahrain* or egypt* or iraq* or jordan* or kuwait* or kuweit* or lebanon or lebanese or libanaise or yemen* or aden or sanaa or UAE or Emirat* or (abu NEAR/2 dhabi) or dubai or libya* or morocco or moroccan* or ifni or (trucial NEAR/2 state) or oman* or muscat or comoros* or palestin* or gaza or ("west bank") or qatar* or katar* or quatar* or saudi* or KSA or Syria* or tunis* or (north* NEAR/2 africa*) or sudan* or djibouti* or somali* or mauritania* or MENA or EMRO or levant or (middle NEAR/2 east*) or ("near east*") or (east* NEAR/2 mediterranean) or orient or arabs or arab or arabia) |
